# Herbal adjuvant therapy with a combination of Green Tea, Persian Borage, and Purslane to reduce antipsychotic-induced weight gain in Schizophrenia: A randomized controlled trial

**DOI:** 10.22038/ajp.2025.26041

**Published:** 2026

**Authors:** Hamideh Naghibi, Mohammad Reza Fayyazi Bordbar, Mahdi Yousefi, Majid Khadem-Rezaiyan, Mohammad Reza Ghanbarzadeh, Seyed Kazem Farahmand, Roshanak Salari

**Affiliations:** 1 *Department of Persian Medicine, School of Persian and Complementary Medicine, Mashhad University of Medical Sciences, Mashhad, Iran*; 2 *Psychiatry and Behavioral Sciences Research Center, Mashhad University of Medical Sciences, Mashhad, Iran *; 3 *Department of Community Medicine, School of Medicine, Mashhad University of Medical Sciences, Mashhad, Iran*; 4 *Department of Persian Medicine, School of Persian and Complementary Medicine, Sabzevar University of Medical Sciences, Sabzevar, Iran*; 5 *Department of Chinese and Complementary Medicine, School of Persian and Complementary Medicine, Mashhad University of Medical Sciences, Mashhad, Iran*; 6 *Department of Pharmaceutical Sciences in Persian Medicine, School of Persian and Complementary Medicine, Mashhad University of Medical Sciences, Mashhad, Iran*

**Keywords:** Green tea, Persian borage, Purslane, Weight gain, Antipsychotic, Schizophrenia

## Abstract

**Objective:**

Second-generation antipsychotics can lead to metabolic problems. This study investigated whether an herbal compound with green tea, Persian borage, and purslane extracts could help in antipsychotic-induced weight management in schizophrenia patients.

**Materials and Methods:**

This triple-blind, placebo-controlled study at Hijazi Psychiatry Hospital in Mashhad, Iran, involved 73 schizophrenia patients. Participants received either an herbal compound or a placebo, alongside their antipsychotic medication. The primary outcome was changes in body mass index (BMI), with secondary outcomes including waist-to-hip ratio (WHR), fasting blood sugar (FBS), HbA1c, lipid profile, blood pressure, appetite, quality of life, and psychotic symptom severity.

**Results:**

The herbal compound significantly reduced BMI (p<0.001), WHR (p<0.001), HbA1c (p=0.042), low-density lipoprotein (LDL) concentration (p=0.009), and systolic blood pressure (p=0.015) compared to the placebo. No significant differences were observed in FBS or lipid profile (except LDL) between the two groups. The intervention group had significantly lower appetite levels than the placebo group at weeks four and eight (p=0.001). There was no significant difference between the two groups in the Positive and Negative Syndrome Scale (PANSS) score at any time. Participants reported no serious adverse effects.

**Conclusion:**

Adding herbal compound to antipsychotics significantly lowered BMI, WHR, HbA1c, LDL levels, systolic blood pressure, and appetite in schizophrenia patients.

## Introduction

Schizophrenia is a psychiatric disease affecting 1% of the population, and its is characterized by positive symptoms (e.g. hallucinations, delusions), negative symptoms (e.g. lack of pleasure or difficulty with speech), and cognitive impairments (Rahman and Lauriello 2016). It is classified among the top ten contributors to the global disease burden by the World Health Organization (Salleh 2018). 

Schizophrenia treatment primarily involves antipsychotic medications, classified as typical and atypical. Atypical antipsychotics or second-generation antipsychotics (SGAs) are preferred due to fewer extrapyramidal effects. Nonetheless, their use is associated with higher metabolic and cardiovascular risks including obesity and reduced life expectancy (Patel et al. 2014). Given the potential side effects and the requirement for long-term use of these medications, patient adherence to treatment may decline (Tschoner et al. 2007). Thus, it is crucial to identify strategies to mitigate these issues. 

Current research demonstrates that medicinal plants serve an essential function in disease management and symptom relief (Javidi et al. 2024; Salehsari et al. 2024). Incorporation of medicinal herbs alongside antipsychotic medications may effectively diminish side effects and enhance the well-being of schizophrenia patients. Utilization of medicinal plants is beneficial owing to their affordability, accessibility, and capacity to tackle various metabolic and psychological complications simultaneously (Naghibi et al. 2023; Shi et al. 2021).

Research indicates that neurochemical alterations, hormonal variations, and sedation-related behaviors lead to heightened appetite and notable weight gain in SGA users (Smith et al. 2012; Stip et al. 2012; Werneke et al. 2013). To enhance pharmacological effectiveness and regulate appetite and weight fluctuations associated with SGAs in schizophrenia, our multidisciplinary research team utilized a synergistic plant-based approach to formulate a novel herbal composition. This new compound consists of herbal extracts of green tea, Persian borage, and purslane for weight control in schizophrenia patients.

Green tea, from Camellia sinensis L., comprises several phytochemicals such as catechins and theanine, which possess antioxidant and anti-inflammatory characteristics (Rani et al. 2014). It may help to prevent weight gain and obesity by reducing intestinal fat absorption, enhancing fat oxidation, increasing energy expenditure (Hursel et al. 2011; Macêdo et al. 2023; Rani et al. 2014; Suzuki et al. 2016; Yang et al. 2016), and improving metabolic parameters, insulin sensitivity, and glucose level (Liu et al. 2013; Wan et al. 2024; Xu et al. 2020). It promotes cardiovascular health through improved blood pressure and endothelial function (Onakpoya et al. 2014; Potenza et al. 2007; Yıldırım Ayaz et al. 2023). Green tea may also suppress appetite via increased dopamine and serotonin levels (Sirotkin and Kolesárová 2021; Zheng et al. 2004). Furthermore, L-theanine in green tea enhances cognitive function and promotes tranquility by regulating neurotransmitters (Hidese et al. 2019; Lardner 2014; Mancini et al. 2017). Green tea shows potential as an adjunctive treatment for schizophrenia and those receiving SGAs (Loftis et al. 2013; Razavi et al. 2017; Ritsner et al. 2011).

Persian borage (Echium amoenum L.), an Iranian medicinal herb, contains various compounds like flavonoids and essential oils that give it anti-inflammatory, antioxidant, anxiolytic, and antidepressant properties (Bekhradnia and Ebrahimzadeh 2016; Miraj and Kiani 2016). Green tea is used in traditional medicine for respiratory, inflammatory, and psychological issues (Azizi et al. 2018; Jafari et al. 2018; Ramezani et al. 2020). Persian borage can prevent inflammatory disorders, regulate dopamine and serotonin levels (Rabbani et al. 2004; Saiiah bargard et al. 2005), lower glucose and lipid levels (Mahmoudi et al. 2015), reduce oxidative stress in the nervous system (Kh and Siddiqui 2024; Sultana et al. 2022), lower blood pressure through its alkaloids and flavonoids and show cardioprotective effects (Roy et al. 2022; Zarshenas et al. 2016). It also has anti-obesity effects by inhibiting pancreatic lipase activity, increasing energy expenditure, promoting fat oxidation, and improving lipid profiles (Abed et al. 2012; Kang et al. 2018; Mahboob et al. 2023). 

Purslane (Portulaca oleracea L.), a nutritious vegetable, has omega-3s, vitamins, and minerals, with health benefits like heart disease prevention, and is approved for obesity or diabetes management (Iranshahy et al. 2017; Li et al. 2024; Srivastava et al. 2023). Its antioxidants and anti-inflammatory properties combat oxidative stress and inflammation (Milkarizi et al. 2024), improve insulin sensitivity (Jafari et al. 2023; Jung et al. 2021), regulate appetite (Cho et al. 2019; Golzar et al. 2023), and improve blood lipids (Azizah et al. 2022; Hadi et al. 2019; Sabzghabaee et al. 2014). Purslane lowers blood glucose by boosting insulin, aiding glucose transport, reducing inflammation, offering polysaccharides and fiber, and protecting against oxidation, making it a valuable addition to combat obesity and diabetes (Ebrahimian et al. 2022; Jung et al. 2021). A recent study suggests that omega-3 supplements can help to improve symptoms of schizophrenia (Hsu and Ouyang 2021). 

Therefore, purslane may be beneficial for SGA-related metabolic changes in schizophrenia patients. 

This study examined the effects of an herbal compound made from green tea, Persian borage, and purslane on weight gain and obesity in schizophrenia patients treated with second-generation antipsychotics at Hijazi Psychiatry Hospital in Mashhad, Iran.

## Materials and Methods

### Study design

The present triple-blind study tested a new herbal compound's effects on weight gain and obesity in schizophrenia patients. All patients, researchers, and data analysts were blinded to the study conditions. It was registered in the Iranian Clinical Trials Registry (IRCT 20210707051813 N1), and conducted from 2021-2023 in Mashhad psychiatric center, Iran. 

### Preparation of medication

Three plants - Camellia sinensis L., Echium amoenum L.*,* and Portulaca oleracea L. - were collected with approval from Mashhad Faculty of Agriculture and Plant Sciences. The herbarium at the Ferdowsi University of Mashhad validated the botanical specimens. Voucher specimen numbers are E-1335 FUMH for C. sinensis L.*,* E-1334 FUMH for E. amoenum L.*,* and E-1248 FUMH for P. oleracea L. After drying, the plants were macerated in 70% ethyl alcohol for 48 hr, then filtered and extracted into dry extracts. Oral herbalcapsules were formulated with 350 mg C. sinensis L. leaves extract, 225 mg E. amoenum L. flowers extract, and 125 mg P. oleracea L. 

Aerial parts extract. The appropriate dosages for each extract were determined from relevant books, reliable sites, and recent articles (Chen et al. 2016; Drugs.com 2022a; Drugs.com 2022b; Duke et al. 2002; Ghahremanitamadon et al. 2014; Girard and Vohra 2011; Huang et al. 2018; Jafari et al. 2023; Jamilian et al. 2014; Lin et al. 2020; Loftis et al. 2013; Nouri et al. 2019; Razavi et al. 2017; Ritsner et al. 2011; Sabzghabaee et al. 2014; Sayyah et al. 2006; Zahedi et al. 2004; Zamansoltani et al. 2008). Based on these references, safe daily dosages for various purposes of extracts are established: green tea extract at 300-1500 mg/dayPersian borage extract at 200-4000 mg/day, and purslane extract at 300-800 mg/day. Considering the risks of liver and kidney damage for SGA consumers, the research team, consisting of a psychiatrist, pharmacist, and herbal medicine specialists, revised the dosages to 700 mg/day for green tea, 500 mg/day for Persian borage, and 250 mg/day for purslane.

Avicel was employed in identical quantity as the herbal capsules to create placebo capsules.

### Standardization of herbal extract capsules

The phenolic compounds in herbal capsules were measured by diluting the product, mixing it with reagents, and assessing the mixture after 2 hr using a spectrometer. The herbal capsule contained 330 mg of gallic acid equivalent per gram of total phenols.

### Participants

The current study focused on schizophrenia patients aged 18-65 with a BMI of 25-40 kg/m^2^ at Hijazi Psychiatry Hospital of Mashhad, Iran, who were admitted during the period from 2021 to 2023. They were on SGAs (including one or more of risperidone, olanzapine, clozapine, or quetiapine) without dosage changes for at least three months and were in the remission phase.

The ineligibility criteria were being patients with serious systemic illness, substance addiction (except nicotine), pregnancy or lactation, liver test elevations (twice the upper limits of normal and more), allergies to herbal medications, or those taking aripiprazole. Dropouts might result from allergic reactions, gastrointestinal intolerance, worsening symptoms, increased blood pressure, or unwillingness to continue participation.

### Intervention

 This placebo-controlled trial studied eligible patients randomly assigned to intervention or placebo groups. The intervention group received herbal extract capsules capsules twice daily for eight weeks as a supplement to their antipsychotic regimen. Herbal extract dosages were safe, chosen based on similar studies. Liver function, and urea, and creatinine levels were monitored for potential adverse effects. The control group received identical capsules containing Avicel. Patients were assessed at baseline and weeks four and eight.

### Randomization and allocation

The present study used a triple-blind approach, using letters A and B to blind participants, researchers, and data analysts. It employed permuted block randomization, with a software-generated unique code concealing the identity of subjects during the randomization process. Sixteen blocks were created to randomize a sample size of 64.

### Outcome measurements

The primary outcome was BMI (kg/m^2^). Secondary outcomes included the assessment of waist-to-hip ratio (WHR) and blood biochemistry analyses. These analyses encompassed fasting blood sugar (FBS), HbA1c, lipid profile, blood urea nitrogen (BUN), creatinine, alanine transaminase (ALT), and aspartate transaminase (AST). Measurements were conducted at baseline and repeated at the end of week eight.

Appetite levels, the severity of psychotic symptoms, and blood pressure (using the Visual Analog Scale (VAS), the PANSS, and a device mercury manometer, respectively) were evaluated at baseline and weeks four and eight, while the quality of life was assessed with the WHOQOL-Bref questionnaire at baseline and week eight.

The WHOQOL-BREF is a 26-item questionnaire that assesses physical and psychological health, social relationships, and environmental health (Wong et al. 2018). It is a reliable test for evaluating the quality of life in schizophrenia patients (Dong et al. 2019; Mas-Expósito et al. 2011) and has been validated in Iran (Masoomi et al. 2018).

PANSS is a widely used tool to assess schizophrenia symptoms, comprising three subscales: Positive, Negative, and General Psychopathology, each rated on a scale of 1 to 7. The total score is the sum of all three subscales. Lower scores indicate milder symptoms, while higher scores suggest more severe symptoms (Smith 2023). The Persian version of this questionnaire is reliable and valid, with a Cronbach's alpha coefficient of 0.83 and 0.77 from two studies, and its validity was confirmed by factor analysis (Abolghasemi 2007; Ghamari Givi et al. 2010).

The visual analog scale is a validated measure of appetite in which subjects rate their appetite levels on a scale of 0 to 10. It was developed by Flint et al. at the Fredrik Berg School of Nutrition in Denmark (Flint et al. 2000). The VAS instrument has been employed in different domains in research on schizophrenia patients (Carrasco-Picazo et al. 2023; Medeiros-Ferreira et al. 2013).

### Sample size calculation

The sample size was determined utilizing a formula for comparing BMI in two groups with a power of 80 % and an alpha error of 5 %, resulting in 64 samples enrolled after accounting for a 10% dropout rate based on a study by Nasri and Taghian (Nasri and Taghian 2020). 

### Statistical analysis

Statistical analysis was conducted utilizing SPSS version 26 software to analyze quantitative and qualitative variables in the study. Descriptive statistics such as mean, standard deviation, frequency, and percentage were employed. The normality of the data was checked through the Kolmogorov–Smirnov test, while baseline characteristics were assessed using chi-square and t-tests for categorical and continuous variables, respectively. Within-group differences were compared using paired t-tests or Wilcoxon tests, and between-group analyses were done with independent sample t-tests or Mann-Whitney u-tests. ANOVA and analysis of covariance (ANCOVA) were utilized to identify differences in means and account for initial values. All tests were two-sided with a significance level set at less than 0.05.

## Results

**Figure 1:**
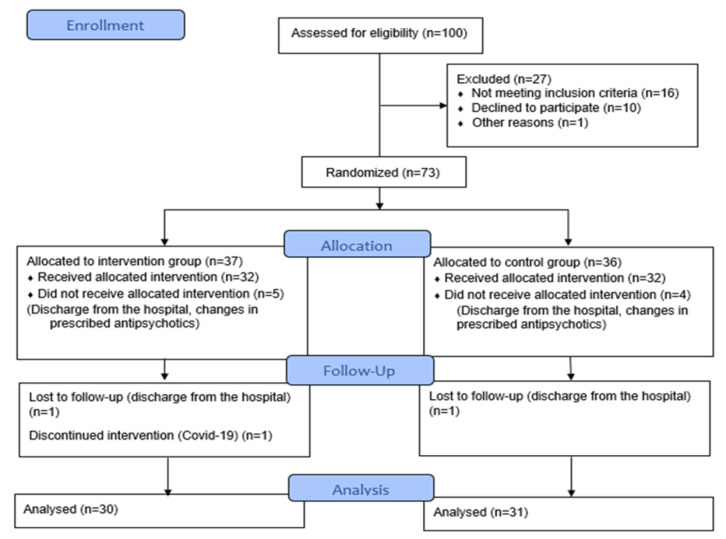
CONSORT Flow Diagram of the study

Seventy-three patients were initially qualified to participate in the study, but the number decreased due to changing medications, or getting COVID-19 (Figure 1). At the end of the 8-week investigation, 61 patients completed the trial; 9 (14.8%) were females, and 52 (85.2%) were males. They were randomly assigned to the intervention group (n=30) and the placebo group (n=31) for eight weeks. The average age of the patients was 44.23 ± 8.03 years. The two groups had no significant differences regarding age, gender, or BMI (p=0.498, p=0.731, and p=0.129, respectively). Additionally, there were no significant differences between the two groups regarding the duration of disease, smoking, physical activity, type of antipsychotics used, or any medication regimen (Table 1). 

In the intervention group, there was a significant decrease in BMI (p<0.001), WHR (p<0.001), total cholesterol levels (p=0.03), LDL concentration (p=0.009), and systolic blood pressure (SBP) (p=0.037) after eight weeks. Additionally, there was a notable increase in high-density lipoprotein (HDL) concentration (p=0.02). The mean AST level significantly increased from 18.43±4.94 to 21.37±5.81 (IU/L) (p=0.002) and the ALT level increased from 19.33±8.76 to 21.73±8.67 (IU/L) (p=0.008) after eight weeks in the intervention group. In the placebo group, the mean level of AST and ALT showed significant changes (p=0.04 and p=0.037), while the other measured variables did not change significantly in this period (for all p>0.05).

**Table 1:** Baseline characteristics of the participants.

	**Intervention (n=30)**	**Placebo (n=31) **	**Total (n=61)**	**p-value **
Age, years	43.40 ±8.24	45.03±7.88	44.23 ± 8.03	0.498 ^d^
Gender, male	25 (83.3 %)	27 (87.1 %)	52 (85.2 %)	0.731 ^b^
BMI (kg/m^2^)	28.13 ± 2.07	28.91 ± 1.86	28.53 ± 1.99	0.129 ^d^
Duration of disease (years)				
< 5 5-10 >10	5 (16.7 %) 16 (53.3 %) 9 (30.0 %)	5 (16.1 %) 13 (41.9 %) 13 (41.9 %)	10 (16.4 %) 29 (47.5 %) 22 (36.1 %)	0.289 ^c^
Smoking	15 (50 %)	14 (45.2 %)	29 (47.5 %)	0.705^ a^
Sport	16 (53.3%)	16 (51.6 %)	32 (52.4 %)	0.893 ^a^
Antipsychotic regimen				
Olanzapine Clozapine Quetiapine Olanzapine-risperidone Clozapine-risperidone Risperidone-Quetiapine Olanzapine-clozapine Olanzapine-Quetiapine Clozapine-Quetiapine Olanzapine-clozapine-quetiapine	1 (3.3 %) 8 (26.7 %) 1 (3.3 %) 3 (10.0 %) 7 (23.3 %) 3 (10.0 %) 4 (13.3 %) 1 (3.3 %) 1 (3.3 %) 1 (3.3 %)	1 (3.2 %) 7 (22.6 %) 2 (6.5 %) 2 (6.5 %) 9 (29 %) 4 (12.9 %) 2 (6.5 %) 3 (9.7 %) 1 (3.2 %) 0 (0 %)	2 (3.3 %) 15 (24.6 %) 3 (4.9 %) 5 (8.2 %) 16 (26.2 %) 7 (11.5 %) 6 (9.8 %) 4 (6.6 %) 2 (3.3 %) 1 (1.6 %)	0.933 ^a^
Anti-diabetic agents				
Metformin Glibenclamide Pitoze	8 (26.7 %) 3 (9.7 %) 3 (9.7%)	12 (38.7 %) 1 (3.3 %) 0 (0.0 %)	20 (32.8%) 4 (6.5%) 4 (6.5%)	0.316 ^a^ 0.354 ^b^ 0.113 ^b^
Anti-lipid agents				
Atorvastatin Gemfibrozil Fenofibrate	17 (56.7%) 2 (6.7%) 1 (3.3%)	13 (41.9%) 0(0.0%) 0(0.0%)	30 (49.2%) 2 (3.2%) 1 (3.3%)	0.250 ^a^ 0.238 ^b^ 0.492 ^b^
Anti-hypertensive agents	3 (10.0%)	6 (19.3%)	9 (14.7%)	0.473 ^b^
Cardiovascular drugs	2 (6.6%)	0 (0.0)	2 (3.2%)	0.238 ^b^

Data are presented as mean ± standard deviation or number (%). Between group analysis was evaluated as follows: a. Chi-square test; b. Fisher's exact test; c. Mann-Whitney u test; and d. independent sample t-test. The level of significance was less than 0.05. BMI: body mass index.

The between-group analysis at week eight showed that the intervention group had significantly lower BMI, WHR, HbA1c, and LDL concentration, and SBP than the placebo group. However, the mean AST level was higher in the intervention group than the placebo group (Table 2).

**Figure 2:**
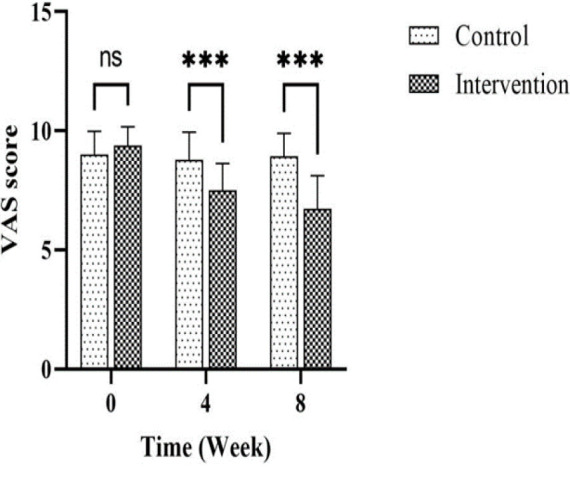
The effect of herbal compound on the appetite level of patients with a visual analog scale. Data are displayed as Mean±SD. Two-way Repeated Measures ANOVA (mixed model) and Bonferroni's post hoc test were used for statistical analysis. ns, Non-significant; ***p>0.001 compared to the control group.

The intervention group had significantly lower VAS scores than the placebo group at weeks four and eight (Table 3 and Figure 2). There were no significant differences between the groups regarding positive, negative, or psychological symptoms and the total score of PANSS at any time point. The Quality of Life scores showed differences in psychological and environmental health dimensions at week eight (Table 3). Smoking status and physical activity remained unchanged for both groups. No significant adverse effects were reported.

**Table 2:** Changes in BMI, anthropometric and clinical indicators, and biochemical parameters in intervention and placebo groups over time.

	** Intervention (n=30)**				**Placebo (n=31)**				**p-value
	**Baseline **	**Week 8**	**Mean difference **	*p-value	**Baseline **	**Week 8**	**Mean difference**	*p-value	
BMI (kg/m^2^)	28.13±2.07	27.13±1.98	-1.00	<0.001	28.91 ± 1.86	29.00±1.75	0.09	0.218	<0.001
WHR	1.01±0.07	1.00±0.07	-0.01	<0.001	1.01±0.08	1.01±0.08	0	0.36	<0.001
HbA1c (%)	5.77±0.57	5.72±0.58	-0.05	0.202	5.92±0.57	6.00±0.58	0.07	0.172	0.042
FBS (mg/dl)	88.23±18.24	87.93±15.97	-0.30	0.81	80.65±11.6	81.23±13.02	0.58	0.756	0.750
TG (mg/dl)	172.73±72.5	165.77±67.6	-6.96	0.12	119.65±50.7	120.94±56.9	1.29	0.762	0.504
Chol (mg/dl)	164.5±33.4	156.17±31.3	-8.33	0.03	136.94±21.01	139.81±25.02	2.87	0.283	0.215
LDL (mg/dl)	99.03±17.53	91.17±18.98	-7.87	0.009	86.77±22.98	88.35±25.18	1.58	0.38	0.021
HDL (mg/dl)	35.57±5.94	37.77±7.05	2.20	0.02	36.55±6.92	38.23±6.65	1.67	0.13	0.870
AST (IU/L)	18.43±4.95	21.37±5.82	2.93	0.002	21.71±6.03	20.03±6.52	-1.68	0.04	0.006
ALT(IU/L)	19.33±8.76	21.73±8.67	2.40	0.008	18.06±7.11	19.52±8.11	1.45	0.037	0.359
BUN (mg/dl)	22.73±7.52	21.80±4.31	-0.93	0.439	23.42±7.00	25.71±10.41	2.29	0.184	0.06
Cr (mg/dl)	0.99±0.13	0.95±0.13	0.04	0.053	0.97±0.11	0.97±0.17	0. 0	0.888	0.394
SBP (mmHg)	120.50±12.13	118.50±11.23	-1.066	0.037	121.29±14.60	121.13±13.70	-0.161	0.879	0.015
DPB (mmHg)	74.00±6.61	73.83±8.06	-0.167	0.920	72.58±9.11	73.71±7.84	1.129	0.467	0.475

Data are presented as mean ± standard deviation; BMI, body mass index; WHR, ratio of waist circumference to hip circumference; HbA1c, glycosylated hemoglobin; FBS, fasting blood sugar; TG, triglycerides; Chol, total cholesterol; HDL, high-density lipoprotein; LDL, low-density lipoprotein; BUN, blood urea nitrogen; Cr, creatinine; ALT, alanine aminotransferase; AST, aspartate aminotransferase; SBP, systolic blood pressure; DBP, diastolic blood pressure. Within-group analysis with Paired-samples t-test; **Between-group analysis with General linear model (multivariate) test. *The level of significance was less than 0.05.

**Table 3:** Changes in VAS score, PANSS score, and WHOQOL – BREF score in the intervention and placebo group at two or three time points.

	**Intervention (n=30)**	**Placebo (n=31)**	**p-value**
**VAS score **			
Baseline Week 4 Week 8	9.47±0.68 7.50±1.11 6.73±1.39	9.00±1.00 8.77±1.18 8.94±0.96	0.58 **<0.001** **<0.001**
**PANSS score **			
*Positive score* Baseline Week 4 Week 8	25.0± 6.86 24.33± 6.69 24.23± 6.81	23.48± 5.36 23.48± 5.26 23.0± 5.1	0.340 0.583 0.428
*Negative score* Baseline Week 4 Week 8	28.57± 6.12 28.67± 5.91 28.60± 5.75	27.84± 5.05 27.42± 4.87 27.29± 4.88	0.614 0.372 0.341
*General score* Baseline Week 4 Week 8	62.83± 5.85 61.0± 6.42 60.53± 6.56	63.81± 5.90 63.16± 6.04 62.97± 6.06	0.521 0.181 0.138
*Total score* Baseline Week 4 Week 8	116.40±12.65 114.00±13.14 113.37±13.06	115.13±11.73 114.06±11.34 113.26±11.76	0.685 0.984 0.973
**WHOQOL – BREF score**			
*Physical health* Baseline Week 8	27.62± 14.22 28.33± 14.43	23.50± 12.63 24.07± 12.12	0.236 0.217
*Psychological health * Baseline Week 8	45.41± 9.68 52.08± 10.36	39.9± 11.87 44.62± 12.13	0.053 **0.012**
*Social relationships * Baseline Week 8	22.50± 19.34 26.38± 21.11	23.92± 18.22 23.65± 19.13	0.768 0.326
*Environmental health* Baseline Week 8	36.14± 7.18 36.14± 6.54	32.15± 8.62 31.85 ± 7.88	0.055 **0.02**
*General health * Baseline Week 8	53.33± 15.02 53.3± 15.02	49.19± 15.79 49.19± 15.79	0.260 0.260

Data were presented as mean ± standard deviation. VAS, Visual Analog Scale; PANSS, Positive, and Negative Syndrome Scale; WHOQL-BREF, World Health Organization Quality of Life Scale. Between-group analysis with independent sample t-test or its nonparametric equivalent (Mann-Whitney u test). The level of significance was less than 0.05.

## Discussion

The present clinical trial tested an herbal combination, as an adjunct for weight control in schizophrenia patients. Results showed reduced BMI, WHR, cholesterol, HbA1c, systolic blood pressure, and appetite and improved HDL levels combined with antipsychotic treatment. 

Antipsychotics are known to cause weight gain by increasing appetite, with the exact mechanism being still unclear. Serotonin receptors play a role in hyperphagia and weight gain induced by SGAs like olanzapine, clozapine, quetiapine, and risperidone, impacting obesity. Olanzapine and clozapine bind to 5-hydroxytryptamine receptor 2a (5HT2a) and 2c receptors, quetiapine blocks 5HT receptors, while risperidone inhibits 5HT2c receptors affecting appetite regulation (Mukherjee et al. 2022).

Research indicates that bioactive compounds in plants such as green tea, Persian borage, and purslane can positively affect metabolism and appetite, potentially offering an effective approach to managing weight gain associated with SGAs (Azizah et al. 2022; Golzar et al. 2023; Hasegawa et al. 2003; Hibi et al. 2018; Huang et al. 2014; Mahmoudi et al. 2015; Rains et al. 2011; Sabzghabaee et al. 2014; Uddin et al. 2014b). 

The protective effects of green tea aqueous extract (GTAE) on olanzapine-related-metabolic changes were studied by Razavi et al. (Razavi et al. 2017). Their study on Wistar rats found that GTAE (25, 50, and 100 mg/kg/day, intraperitoneally.) significantly reduced body weight gain, improved lipid profile, and blood glucose, and lowered hyperleptinemia and hypertension. In a clinical study, Loftis et al. found that 150 mg of epigallocatechin gallate (EGCG) did not significantly decrease psychiatric symptoms in schizophrenia and bipolar disorder over 8 weeks (Loftis et al. 2013). In contrast, Ritsner et al. discovered that 400 mg/d of L-theanine improved positive symptoms, activation, and anxiety in patients with schizophrenia/schizoaffective disorder (Ritsner et al. 2011). Neither of these two studies examined metabolic changes or weight.

The antidepressant and anxiolytic effects of Echium amoenum have been found in studies, but research on its impact on psychosis is limited. A study by Sayyah et al. (Sayyah et al. 2006) using 375 mg of Echium amoenum aqueous extract reduced depressive symptoms. However, our study did not observe significant changes in patients' negative or positive symptoms. Zamansoltani et al. (Zamansoltani et al. 2008) found that administering an aqueous extract of Echium amoenum (100, 200, or 400 mg/kg/day) decreased serum levels of ALT and Alkaline phosphatase (ALP) in rats, while Zahedi et al. (Zahedi et al. 2004) showed that a methanolic extract of Echium amoenum (200 mg/kg) increased ALT and AST serum levels. 

Purslane has the highest levels of omega-3 fatty acids among plants (Uddin et al. 2014a). Recent research suggests that schizophrenia patients lack omega-3 fatty acids, with supplementation improving positive and negative symptoms (Hsu and Ouyang 2021; Jamilian et al. 2014). In the Jamilian et al. study, 1000 mg/day omega-3 supplementation decreased the general psychopathologic score in PANSS in 60 schizophrenia patients (Jamilian et al. 2014). 

In similar studies, a singular plant was analyzed; conversely, our research employed a triad of plants for enhanced efficacy. The synergistic interaction of these three plants may facilitate weight management. The catechins and caffeine present in green tea are posited to elevate metabolism and fat oxidation, while Persian borage and purslane contribute to inflammation management, nutrient absorption, and satiety enhancement. This integrative strategy may promote healthier weight by addressing energy expenditure and caloric intake. Moreover, the enhancement of insulin sensitivity, inflammation reduction, antioxidant properties, and the omega-3 and nutrient density of the herbal compound may aid in metabolic syndrome management.

Our research found that taking 700 mg of this herbal extract twice daily for eight weeks helped to manage weight gain in schizophrenia patients by controlling appetite and reducing cholesterol. Furthermore, herbal formula significantly decreased HbA1c levels within eight weeks. While it is ideal to compare changes in HbA1c levels over 12 weeks, the HbA1c test is more reliable than FBS for evaluating serum glucose levels due to its resistance to short-term influences like food intake, exercise, or stress.

Additionally, giving 700 mg of hydroalcoholic herbal extracts twice daily raised AST and ALT serum levels by 2-3 IU/L over eight weeks, within normal ranges. Still, caution is advised due to potential hepatotoxicity. Administering a safe dose of herbal medications is crucial to prevent liver issues and worsening of symptoms, although future studies should use more thorough tests to assess liver function.

The research sought to utilize herbal compounds for weight management and psychiatric symptom relief associated with SGAs. Nonetheless, the herbal compound did not demonstrate notable enhancements in patients' quality of life or PANSS scores. Given dopamine's role in pleasure and motivation, it was hypothesized that the herbal compound could enhance patient outcomes by modulating dopamine levels. However, the limited results may stem from concerns regarding the side effects of higher dosages and the study's duration.

The herbs we utilized could be effortlessly integrated into the patients' diet as decoctions, herbal tea, or in food recipes.

Twice-daily use of herbal capsule, a compound of green tea, Persian borage, and Purslane extract, with antipsychotics can help manage weight in schizophrenia patients. It is recommended to combine these extracts with a balanced diet and regular exercise for best results.
